# Standard operating procedures for biobank in oncology

**DOI:** 10.3389/fmolb.2022.967310

**Published:** 2022-08-26

**Authors:** Giuseppina Bonizzi, Lorenzo Zattoni, Maria Capra, Cristina Cassi, Giulio Taliento, Mariia Ivanova, Elena Guerini-Rocco, Marzia Fumagalli, Massimo Monturano, Adriana Albini, Giuseppe Viale, Roberto Orecchia, Nicola Fusco

**Affiliations:** ^1^ Biobank for Translational and Digital Medicine, IEO, European Institute of Oncology IRCCS, Milan, Italy; ^2^ Department of Oncology and Hemato-Oncology, University of Milan, Milan, Italy; ^3^ Division of Pathology, IEO, European Institute of Oncology IRCCS, Milan, Italy; ^4^ Technology Transfer Office, IEO, European Institute of Oncology IRCCS, Milan, Italy; ^5^ Patient Safety and Risk Management Service, IEO, European Institute of Oncology IRCCS, Milan, Italy; ^6^ Scientific Directorate, IEO, European Institute of Oncology IRCCS, Milan, Italy

**Keywords:** biobank, translational research, biomarkers, cancer research, tissue samples, liquid biopsy, standard operating procedures, quality control

## Abstract

Biobanks are biorepositories that collect, process, store, catalog, and distribute human biological samples, and record the associated data. The role and action field of these strategic infrastructures for implementing precision medicine in translational research is continuously evolving. To ensure the optimal quality at all stages of biobanking, specific protocols are required and should be elaborated according to updated guidelines, recommendations, laws, and rules. This article illustrates the standard operating procedures, including protocols, troubleshooting, and quality controls, of a fully certified biobank in a referral Cancer Center. This model involves all clinical departments and research groups to support the dual mission of academic cancer centers, i.e. to provide high-quality care and high-quality research. All biobanking activities based on the type of biological specimens are detailed and the most tricky methodological aspects are discussed, from patients’ informed consent to specimen management.

## Introduction

Modern oncologic research requires that high-quality biological samples and the associated data are collected, tracked, processed, stored, cataloged, and distributed to research groups and collaborating partners (Kinkorová, 2015). This integrated biobanking approach has led to breakthroughs in both biomarker discovery and drug development (Pagni et al., 2019). Biobanks thus represent essential resources for basic, translational, and clinical research but they also act as key players linking academic research and the pharmaceutical biotechnology industry (Vaught et al., 2011; Hewitt and Watson, 2013; Jose et al., 2018; Coppola et al., 2019). Moreover, the ability to integrate not only clinical information but also biospecimens into big data repositories has intensified the centrality of biobanks (Margolis et al., 2014; Kinkorová, 2015; Drosou et al., 2017; Kinkorová and Topolčan, 2020). This is especially important as biorepositories have begun to incorporate patient information with comprehensive clinicopathologic, epidemiologic, and demographic data, together with multi-omics molecular information (Braun et al., 2014; Luo et al., 2014; Leff and Yang, 2015; Saifuddin et al., 2017; Bycroft et al., 2018; Hulsen et al., 2019; Bonnechère et al., 2021). The collection of this increasing amount of data requires strict quality controls and standard operating procedures (SOPs). The genomic and post-genomic field area has generated a high demand for high-quality biospecimen and data. Biorepositories in cancer research support scientists and clinicians to obtain disease-specific insights. For these reasons, biobanks should be established and updated following international guidelines, such as those from the International Agency for Research on Cancer (IARC), U.S. National Cancer Institute, United Kingdom. Confederation of Cancer Biobanks, and International Society for Biological and Environmental Repositories (ISBER), recommendations, laws, and rules (Vaught et al., 2009; Sanchini et al., 2016; Mendy et al., 2017). In this respect, networking is essential for sharing materials and data among institutions and research groups, particularly for the study of rare diseases (Montserrat and Taruscio, 2019).

Here, we present the organization of a fully certified (UNI EN ISO 9001:2015 - Certiquality) biobank in a referral Cancer Center, which is an integral part of the Italian node of the European Research Infrastructure on Biobanking (BBMRI-ERIC) (Salvaterra and Corfield, 2017). This facility works in compliance with the new standard ISO 20387:2018 “Biotechnology - Biobanking - General requirements for Biobanks”. All SOPs herein reported fulfilled the BBMRI-ERIC quality control and audits (https://www.bbmri-eric.eu/services/quality-management/). Protocols and best practices for the collection of surgical tissue samples, as well as biofluids (e.g., plasma, serum, blood, urine), cell cultures, and peripheral blood mononuclear cells (PBMC), are described in detail (Kanof et al., 2001; Elliott and Peakman, 2008; Guerin et al., 2010; Mallone et al., 2011; Fisher et al., 2018; Hojat et al., 2019; Rolfo et al., 2021). This model enables collaboration among research groups and industry, allowing patients to be an integral part of translational research.

## Scientific/ethical approval and patients’ recruitment

All procedures involving biobanks must be approved by the local Scientific and Technical Committee, the Ethics Committee, and the directors of the involved clinical Units and surgical programs, according to the 1964 Declaration of Helsinki, the 2018 General Data Protection Regulation (GDPR), and subsequent amendments (Sanchini et al., 2016). In this prototype, the GDPR is represented by the Scientific Research Participation Agreement (SRPA), which is the standard informed consent that patients sign to donate biological samples, sensitive data, or genetic data at the European Institute of Oncology in Milan, Italy. The SRPA should be obtained from all patients for the storage, processing, and use of the data obtained for scientific purposes. Only the signed SRPA allows for biospecimen collection. Through this agreement, each patient can express his or her will and modalities of scientific research participation. Given the complexity of the concepts described in the SRPA, it is advisable to share educational material with the patients. For example, as a reminder of their first visit, patients can receive a text message whereby the SRPA information is provided. In addition, short engaging videos broadcasted in the waiting rooms can be employed to inform patients about the importance and implications of the SRPA. An example of a cartoon on biobank used at the European Institute of Oncology, Milan, Italy is freely available online (https://vimeo.com/679070846). During each phase of the hospitalization, SRPA can be administered to the patient by qualified professionals, including biobank staff, nurses, physicians, and biomedical personnel. If patients agree to participate in any study, they should receive all the specific study information approved by the Ethics Committee. Informed consent in the form of SRPA is obtained from all patients for their material to be stored in the biobank and used for further studies. Hence, SOPs, guidelines, and recommendations do not permit the collection and storage of biospecimens in the absence of patients’ consent. Therefore, SRPA should be continuously updated to inform patients properly and comprehensively. To solve any patient’s withdrawal from the previous SRPA, a patient sample take-out methodology should be implemented (Schmanski et al., 2021). To obtain the collected samples, researchers, or external collaborators (for-profit or non-profit) should apply to the Biobank Scientific-Technical Committee and/or the Institutional Ethics Committee. A specific form has to be filled by the applicant for evaluation and linked to an approved evaluation of the project.

## Management of biological samples

After previous verification of the patients’ SRPA, each biological sample can be collected and treated. Samples may include: fresh and frozen tissue samples related to the patients who underwent surgery; fresh and frozen tissue biopsies; cytological samples (e.g., needle aspirations, excreted, ascites, pleural fluids) from needle aspiration or brushing/scraping, and from affixing to surgically removed tissues for small lesions; biofluids (blood, serum, plasma, PBMC, oral swab, urine, feces, ascites) of patients in pre-hospitalization, patients enrolled in clinical trials, and any other subjects involved in screening projects. Each phase of samples collection should be compliant with the latest ISO standards (e.g. ISO 20387:2018).

### Check-in, aliquoting, and distribution

**FIGURE 1:**
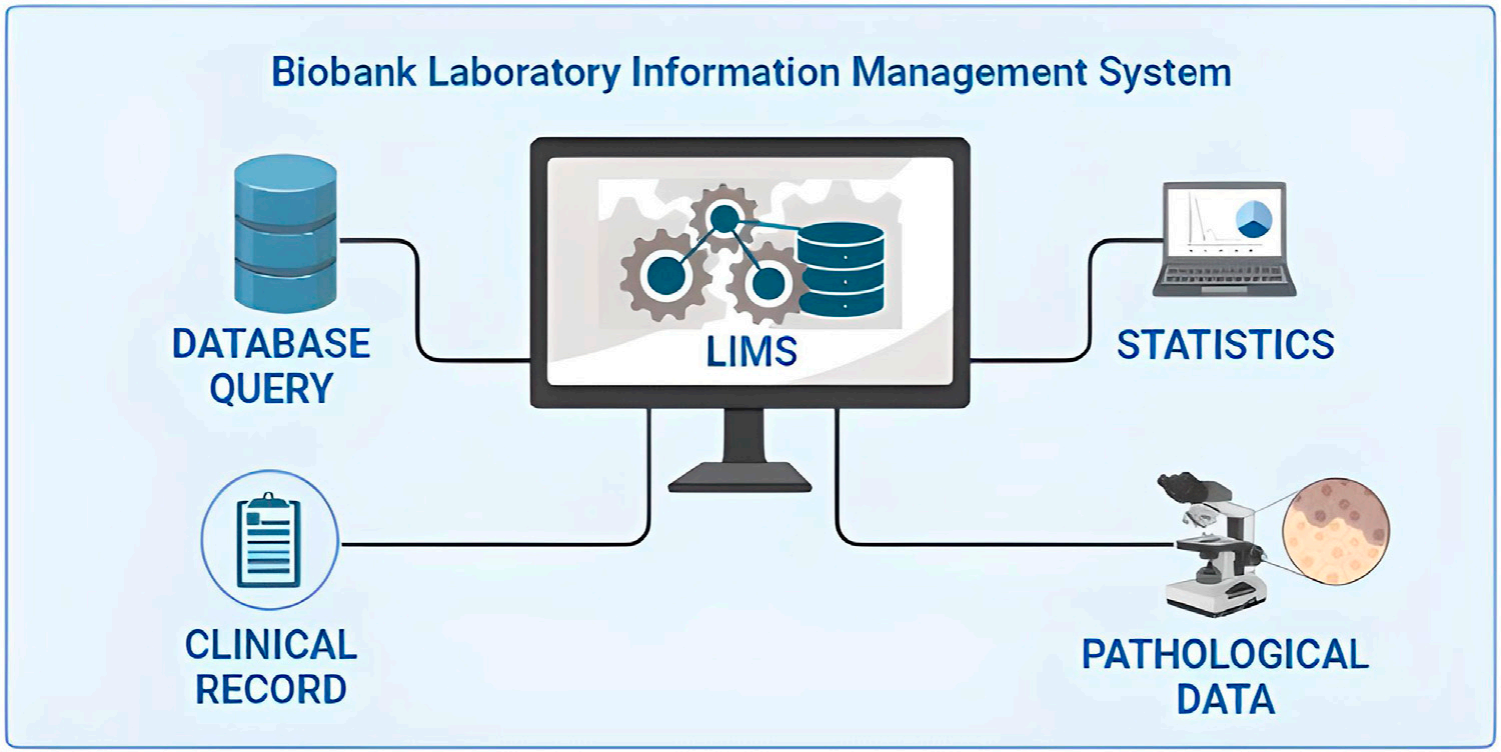
Integration of the laboratory information management system (LIMS) of biobanks in the critical junction of data from different sources.

**FIGURE 2:**
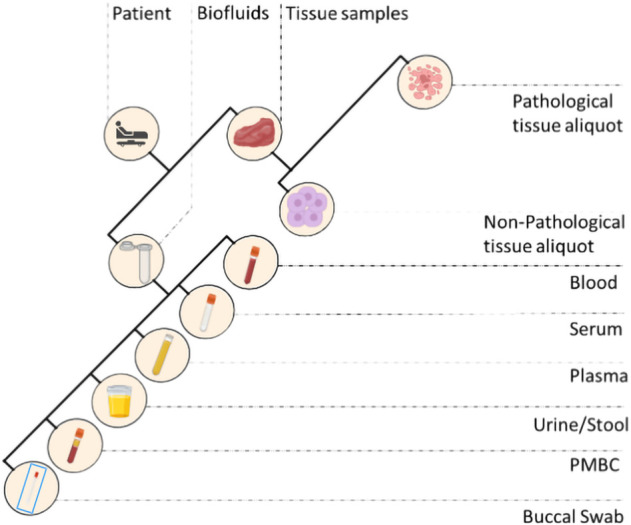
Different types of biospecimens collected in a standard biobank. PMBC, peripheral blood mononuclear cells.

When surgical intervention is scheduled, the Biobank data manager should check in the Surgery Plan if the patient is eligible for samples collection by the signed SRPA presence. If eligible, a surgery plan for biobank must be prepared to check the correspondence of the patient’s inclusion/exclusion criteria, and the patient’s consent for clinical trial or research project. To store and track the significant amount of data generated from the processing and analysis of patients’ biological samples, the use a Laboratory Information Management System (LIMS) software named is highly recommended. This software is considered the biobank neural network because it should ideally interact bidirectionally with all the other softwares and applications used in the Institution, as portrayed in Figure 1. All processing and analysis are tracked by the LIMS, allowing the biobank operators to minimize possible errors, as each type of aliquot is electronically recorded using barcodes (Figure 2). Further, all samples should be divided into subcategories according to the processing, such as the fresh sample, and the frozen sample. The aliquots’ ID is a sequential series of numbers and letters generated by LIMS, according to SOPs, as exemplified for biofluids in Table 1 and tissue samples in Table 2. An example of an ID code is: “12-B1-00100-01” where “12” indicates the year (2012), “B1” indicates the anatomical origin (left breast), and “00100-01” identifies the sample. Once an aliquot is requested, a database query allows the retrieval of the requested samples. This process needs a tracked form with information, a short description of the approved research project, and information on the principal investigator (PI). Consequently, the LIMS generates the requested ID for each aliquot sorted into a picklist. Later, the biobank technicians can check the ID list to retrieve the requested aliquots. Last, barcoded tubes (e.g. Nunc™ Coded Cryobank Vial Systems, Thermo Scientific, Waltham, Massachusetts, US) and relative data can be delivered.

**TABLE 1:** Examples of non-tissue sample types and corresponding biobank codes.

Matrix	Code
ASCITES	AS
WHOLE BLOOD	BL
BRONCHOSCOPY	BS
BUCCAL SWAB	BU
BUFFY COAT	BC
CYTOLOGICAL SAMPLE, NOS	CY
FECES	F
PERIPHERAL BLOOD MONONUCLEAR CELLS (PBMCs)	PB
BLOOD PLASMA	PL
BLOOD SERUM	SE
TUBE BRUSHING	TB
URINE	U

**TABLE 2:** Examples of tissue sample types and corresponding biobank codes.

Atrix	Code
ABDOMEN, NOS	A
ADRENAL GLAND	AG
BONE TISSUE	BO
BONE MARROW	BM
BRAIN	BR
BREAST, NOS	B
BREAST - LEFT	B1
BREAST - RIGHT	B2
MESENTERY	M
CERVIX	CE
COLON	C
ESOPHAGUS	E
FIMBRIA, NOS	FI
FIMBRIA - LEFT	FI1
FIMBRIA - RIGHT	FI2
KIDNEY	K
ILEUM	I
LARYNX	LA
LIVER	LI
LUNG, NOS	L
LUNG - LEFT	L1
LUNG - RIGHT	L2
LYMPH NODE	LN
LYMPH NODE-ABDOMINAL	LNA
MESENTERY	M
NASOPHARYNX	NA
OMENTUM	OM
ORAL CAVITY	OR
OROPHARYNX	OP
OVARY, NOS	O
OVARY - LEFT	O1
OVARY - RIGHT	O2
PANCREAS	PA
PHARYNX	PH
PERITONEUM	PE
PROSTATE	*p*
RECTUM	R
SKIN	SK
SOFT TISSUES	ST
STOMACH	S
TESTIS	TE
THYMUS	TH
THYROID	T
TONGUE	TO
FALLOPIAN TUBE	TU
URINARY BLADDER	UB
UTERUS CORPUS	UC

### Sample types

#### Tissue

During the gross examination of the surgical sample, the pathologist determines whether there is sufficient material (i.e. exceeding diagnosis) for research purposes. When available/possible, the non-pathological counterpart is also collected. Normal and tumor samples are labelled and placed in sterile Petri dishes on ice and divided into fresh and/or optimal cutting temperature (OCT) compound aliquots. Samples are then frozen for 3 min at −120°C in isopentane before transfer to cryopreservation rooms and stored at −80°C. It is important to perform a quality check for each frozen aliquot before distribution and use it to obtain a histological assessment of the cellularity on hematoxylin and eosin (H&E) cryosections (Table 3).

**TABLE 3:** Representative quality Control Form of tissue sections included in OCT and frozen.

Label with biobank code	—
Tumor Tissue (%)	—
Tumor Tissue Description	—
Normal Tissue Counterpart (%)	—
Normal Tissue Counterpart Description	—
Necrotic Tissue (%)	—
Adipose Tissue (%)	—
Stromal Tissue (%)	—
Inflammation	☐ Absent
	☐ Sparse
	☐ Intermediate
	☐ Extensive
Diameter of Section (mm)	
Conclusion	☐ Insufficient
☐ Sufficient
Notes	—
Technical Operator	—
Pathologist Operator	—

#### Whole blood

For whole blood samples collection, 6 ml labeled vacutainer tubes, containing anti-coagulant Na2 ethylenediaminetetraacetic acid (EDTA), are used by the nurses at the assessment centers. Following the collection, the biobank technicians process the sample using its medical record number and the episode code and register each aliquot in the biobank software. For clinical studies, or project-specific requirements, the blood is prepared for shipment, otherwise it is firstly collected using a blood amount of 900 μL in 1 ml barcoded cryotubes for one or more aliquots, and stored at −80°C.

#### Blood serum

For blood serum samples collection, 6 ml labeled vacutainer tubes containing a thixotropic barrier gel at the bottom of the tube, are initially used. Tubes are left for clotting for 3 h at room temperature (RT), and then centrifugated on a refrigerated centrifuge at 828 x g for 10 min. Depending on the initial amount of whole blood taken, the biobank technicians’ rate 450 μL of serum in the 0.5 ml barcoded cryotubes for the maximum amount of aliquots, and stored at −80°C. When the last aliquot serum volume is < 450 μL, the sample is registered as “leftover”.

#### Blood plasma and cf-DNA/RNA

To separate the plasma from the whole blood, blood-filled vacutainers need to be centrifuged at 2,000 x g for 10 min at RT. After centrifugation, the upper plasma layer should be removed and transferred to a sterile 15 ml conical tube for a second centrifugation at 16,000 x g for 10 min at RT to remove contaminating blood cells. Then, the obtained plasma can be transferred to a barcoded cryotube for the maximum amount of aliquots, depending on the volume. The remaining blood is collected for subsequent cf-DNA and/or cf-RNA purification. Aliquots should be stored at −80°C.

#### Peripheral blood mononuclear cells

For peripheral blood mononuclear cells (PBMC) isolation, the blood is drawn in 7.5 ml labeled vacutainer tubes, containing Na_2_ EDTA as an anticoagulant reagent. The blood is subsequently transferred into an empty 50 ml conical tube and diluted in a 1:1 ratio using phosphate buffered saline (PBS) 1X (e.g., 15 ml of blood +15 ml of PBS 1X). Again, the diluted blood is layered on the top of a clean 50 ml conical tube containing 15 ml of Ficoll, without mixing the two solutions. After centrifugation at 400 x g for 30 min at RT, the white layer containing PBMC is collected and placed in a new sterile 50 ml conical tube. PBMCs are washed by adding 45 ml of PBS, mixed, and centrifuged at 400 x g for 10 min at 4°C. After discarding the supernatant, the pellet containing PBMCs is resuspended in PBS and counted using Tuerks solution and single-use slide for counting cells (e.g. Biosigma S.P.A. Cat. no. 347143/001). Cells are washed once more with PBS and resuspended at 2-3x10ˆ6 cells/mL of FSB +10% dimethyl sulfoxide (DMSO) to be frozen. Finally, 1 ml of resuspended cells are transferred in cryotubes for storage at −80°C.

#### Stool and buccal swab

Feces and buccal swabs are collected in 15 ml tubes (e.g. Stool Sample Collection and Stabilization Kit Canvax Cat. no 0013), containing 50 mM Tris-HCl, 10 mM NaCl, and 10 mM EDTA pH 7.5 and stored at −80°C.

## Disaster recovery plan

Biobanks are dedicated to managing valuable and possibly irreplaceable biological specimens. Therefore, biomaterials and associated data must be managed and protected carefully as their loss can destroy years of research efforts and costs, and potentially result in damage to the Institution (Eng and Tan, 2019). For this reason, risk management and practical crisis management plans must be established for any biobank (Parry-Jones et al., 2017). It is essential to define a data protection program that must satisfy various needs that may range from remote data only (backup) to disaster recovery (as a set of technological measures and organizational processes aimed at restoring systems, data, and infrastructures necessary to provide biobank services during emergencies) and to ensure continuity of service and recovery of materials and data during emergencies (Cicek and Olson, 2020).

## Staff training programs

The quality and quantity of samples and data stored in a biobank directly depend on the biobank personnel, including data managers and technicians (Hartung et al., 2021). Modern biobanking must rely on high-level training programs for biobank employees not only to allow harmonization of correct sample handling but also to ensure safety and quality (Kinkorová and Topolčan, 2020). Not surprisingly, training certificates of biobank employees are needed for the accreditation process (Williams et al., 2019). Types of training programs include master’s programs, certificate courses, and workshops. Due to the paucity of available formal training programs, biobanks often train most of their new staff on site (Castellanos-Uribe et al., 2020). Learning about teamwork, personnel safety, patient privacy, biospecimen quality, and SOPs is crucial not only for efficiency and productivity but also for the personnel’s career success. A well-designed training program should include helpful tips, tricks, and troubleshooting. International collaboration and exchange programs might facilitate the process of creating next-generation biobanking staff.

## Representative results

**FIGURE 3:**
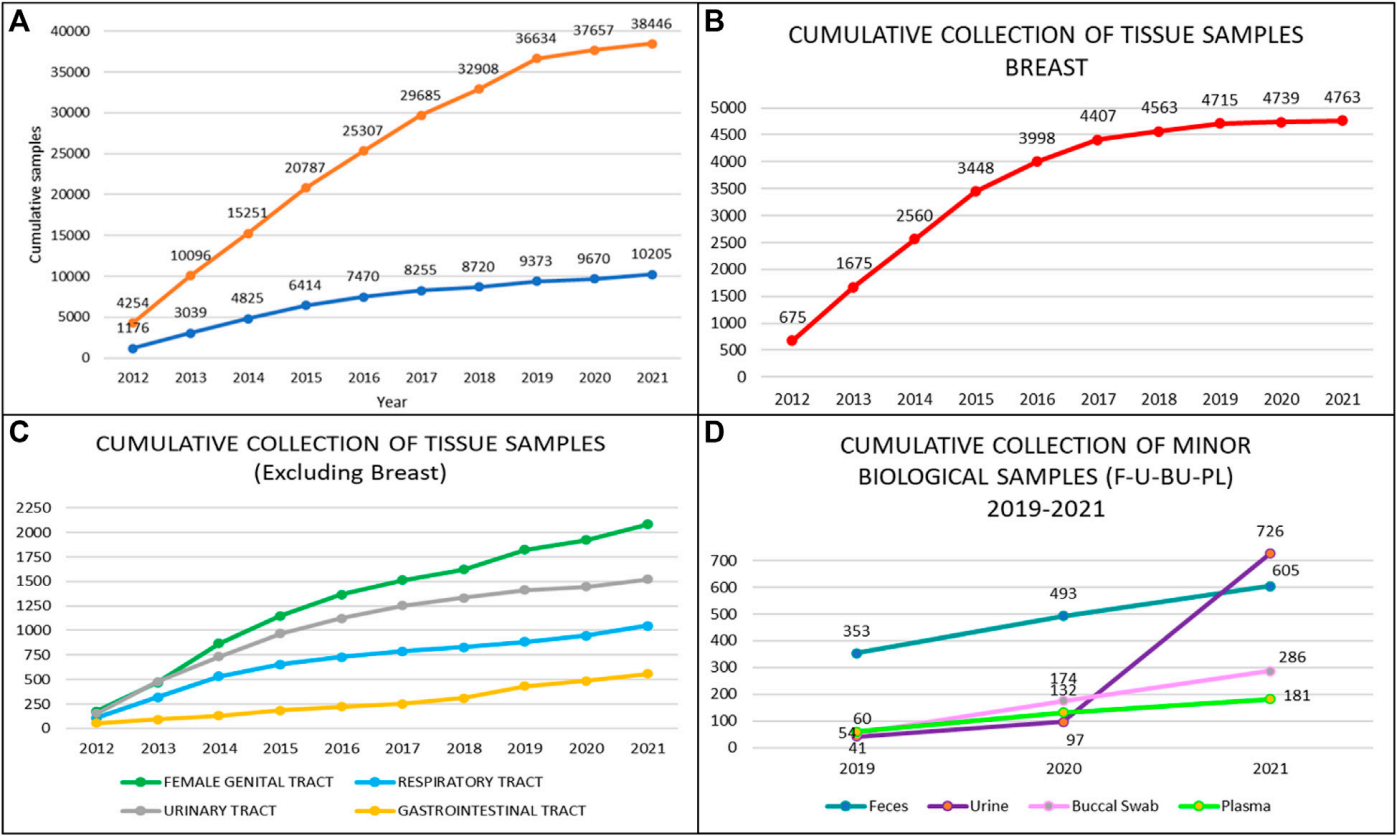
Types and number of samples collected by the biobank of the European Institute of Oncology by year. **(A)** Cumulative collection of tissue and blood/serum samples; **(B)** Cumulative collection of breast tissue samples; **(C)** Cumulative collection of tumor samples from the ovary, prostate, lung, and colon; **(D)** Cumulative collection of non-tissue samples, i.e. feces, saliva/swab, plasma, and urine.

A total of 38,446 annotated biofluids and a total of 10,205 tissue samples were collected by the Biobank for Translational and Digital Medicine Unit at the IEO, European Institute of Oncology, Milan, Italy from April 2012 to December 2021 (Figure 3A). The highest number of samples were related to breast cancer, urological malignancies, tumors of the female genital tract, head and neck carcinomas, and lung cancer (Figures 3B,C). The cumulative analysis of plasma, buccal swab, urine, and stool samples revealed a significant increase in the number of collected samples, particularly for the urine in patients with urological malignancies, reaching a total number of 726 urine samples (Figure 3D). These samples have been divided into multiple aliquots related to specific projects or clinical trials and to the institutional universal collection of samples. Taken together, 28 different projects were responsible for the vast majority of aliquot distribution, for a total number of 28,852 aliquots, as detailed in Table 4. The total number of tissue samples whose aliquots were employed for research purposes were 8,383/10,205 (82%). These data confirm the fundamental role of certified biobanks not only for samples collection but also for samples distribution and use by research groups.

**TABLE 4:** Distribution of samples aliquots from the biobank of the European Institute of Oncology.

Project ID	2012	2013	2014	2015	2016	2017	2018	2019	2020	2021	Total
T-CELL	—	—	—	—	—	—	46	182	144	137	509
BLADDER	—	—	—	—	5	16	—	—	—	—	21
BREAST-1	1,168	1,611	1,340	756	258	65	217	160	12	40	5,627
BREAST-2	—	—	—	—	—	—	26	79	100	65	270
BREAST-3	—	—	—	—	—	—	—	122	122	90	334
COLON-1	—	—	—	—	—	—	22	—	185	—	207
COLON-2	—	—	—	46	85	97	105	37	—	—	370
COLON-3	—	—	—	—	—	—	20	14	13	—	47
COLON-4	—	—	—	—	—	—	—	563	—	—	563
COSMOS	5	14	4	3,000	363	—	—	—	—	—	3,386
FAM	—	—	—	—	164	—	—	—	—	—	164
H&N-1	—	—	—	6	46	22	56	51	20	134	335
H&N-2	—	—	—	—	—	—	—	—	—	35	35
H&N-3	—	—	—	—	—	—	—	—	—	65	65
LUNG-1	246	311	338	103	26	20	—	—	—	—	1,044
LUNG-2	—	—	—	—	—	—	—	80	498	342	920
LUNG-3	—	—	—	—	—	—	186	—	—	—	186
MEL-1	—	—	—	—	—	—	—	—	—	2	2
MEL-2	—	—	—	—	—	—	—	628	752	417	1,797
miRNA	120	117	206	214	50	—	—	—	—	—	707
OVARY-1	320	569	696	561	402	312	204	664	146	166	4,040
OVARY-2							26	113	102	77	318
PROSTATE	275	545	299	200	127	128	181	66	23		1,844
SARCOMA	—	—	—	—	—	—	—	—	—	9	9
SKIN	—	—	—	—	—	—	23	10	—	47	80
STOMACH	—	—	—	—	—	—	—	—	—	193	193
TEST COVID	—	—	—	—	—	—	—		692		692
THYMUS	—	—	—	—	2	19	24	18	11	13	87
**TOTAL**	2,134	3,167	2,883	4,886	1,528	679	1,136	2,787	2,820	1,832	23,852

## Discussion

The transversal role of biobanks in scientific research, particularly in oncologic pathology, and basic and clinical sciences, has put these important infrastructures on the front line of personalized medicine evolution (Kinkorová, 2015; Coppola et al., 2019). Indeed, cancer is still a leading cause of morbidity and mortality worldwide (Sung et al., 2021). For these reasons, the understanding of cancer pathogenesis, mechanisms of disease, and biomarkers discovery at the multi-omics level is becoming an urgent clinical need, akin the support in drug discovery (Kinkorová, 2015; Yan et al., 2018; Szustakowski et al., 2021). When a biobank is established several challenges, from methodological to operative and ethical issues need to be assessed. The first important point is to manage the existing institution data. This can be done by using a laboratory information management system software able to receive and integrate different types of information, from clinical to pathological and digital data. In literature, several valuable softwares have been used to implement biobank databases (Tukacs et al., 2012; Paul et al., 2017; Fthenou et al., 2019; Im et al., 2019), and some freeware can be obtained for biobank management (Voegele et al., 2013; Willers et al., 2021). A biobank consent form is another critical step in data acquisition and management (Beskow et al., 2017; Kinkorová et al., 2019; Kasperbauer et al., 2021; Schmanski et al., 2021). The application of an adequate SRPA is a necessary agreement on legal and ethical aspects of the patient’s data storage and usage (D'Abramo et al., 2015; Sanchini et al., 2016). All SOPs described in this work are continuously under evaluation and improvement and they are currently compliant with the ISO 20387:2018 standards. It should be noted, however, that standardization and improvement of pre-analytical procedures for *in-vitro* diagnostics is a continuous process. The most updated high-priority pre-analytical CEN and ISO standard documents as well as corresponding External Quality Assessment (EQA) schemes and implementation tools are detailed in Table 5. Not only following adequate SOPs is essential to secure research achievements, but also have qualified personnel, aware of the biobank’s role and potential (Caixeiro et al., 2016; Kintossou et al., 2020). Another important aspect related to the multidisciplinary collaboration in biobanking is represented by the pathologist (Angerilli et al., 2021). Pathologists are the only professionals able to ensure both the tissue sampling for diagnosis and the biobank. Finally, it should be mentioned that the efforts and resources invested to set up and sustain a biobank are significant and such work should be traced and, most importantly, recognized in scientific publications (Howard et al., 2018). In this respect, the Bioresource Research Impact Factor/Framework (BRIF) initiative was proposed for transparency and to promote the responsible and effective use of biomaterials (Cambon-Thomsen, 2003). Another point that is worth mentioning is related to the integration of artificial intelligence (AI) and machine learning into modern biobanks (Kinkorová and Topolčan, 2020; Eccher et al., 2021; Narita et al., 2021; Rizzo et al., 2022). This would allow for the integration of a digitalized database with digital pathology and high throughput molecular data, potentially representing a quantum leap in biobanking.

**TABLE 5:** European Committee for Standardization Technical Committee (CEN/TC) 140 *in vitro* diagnostic medical devices published standards**.** All projects are sorted by date and available at https://www.spidia.eu/projects/standard-documents (Accessed 28 July 2022).

References	Date	Title
CEN/TS 17811:2022	22 June 2022	Molecular *in vitro* diagnostic examinations - Specifications for pre-examination processes for urine and other body fluids - Isolated cell free DNA
CEN/TS 17747:2022	20 April 2022	Molecular *in vitro* diagnostic examinations - Specifications for pre-examination processes for exosomes and other extracellular vesicles in venous whole blood - DNA, RNA and proteins
CEN/TS 17742:2022	30 March 2022	Molecular *in vitro* diagnostic examinations - Specifications for pre-examination processes for venous whole blood - Isolated circulating cell free RNA from plasma
EN ISO 20776-2:2022	19 January 2022	Clinical laboratory testing and *in vitro* diagnostic test systems - Susceptibility testing of infectious agents and evaluation of performance of antimicrobial susceptibility test devices - Part 2: Evaluation of performance of antimicrobial susceptibility test devices against References broth micro-dilution (ISO 20776-2:2021)
CEN/TS 17688-2:2021	22 December 2021	Molecular *in vitro* diagnostic examinations - Specifications for pre-examination processes for Fine Needle Aspirates (FNAs) - Part 2: Isolated proteins
CEN/TS 17688-1:2021	22 December 2021	Molecular *in vitro* diagnostic examinations - Specifications for pre-examination processes for Fine Needle Aspirates (FNAs) - Part 1: Isolated cellular RNA
CEN/TS 17688-3:2021	22 December 2021	Molecular *in vitro* diagnostic examinations - Specifications for pre-examination processes for Fine Needle Aspirates (FNAs) - Part 3: Isolated genomic DNA
EN ISO 4307:2021	3 November 2021	Molecular *in vitro* diagnostic examinations - Specifications for pre-examination processes for saliva - Isolated human DNA (ISO 4307:2021)
EN ISO 16256:2021	27 October 2021	Clinical laboratory testing and *in vitro* diagnostic test systems - Broth micro-dilution References method for testing the *in vitro* activity of antimicrobial agents against yeast fungi involved in infectious diseases (ISO 16256:2021)
EN ISO 6717:2021	8 September 2021	*In vitro* diagnostic medical devices - Single-use containers for the collection of specimens from humans other than blood (ISO 6717:2021)
EN ISO 20166-4:2021	28 July 2021	Molecular *in vitro* diagnostic examinations - Specifications for preexamination processes for formalin-fixed and paraffin-embedded (FFPE) tissue - Part 4: *In situ* detection techniques (ISO 20166-4:2021)
EN ISO 23162:2021	14 July 2021	Basic semen examination - Specification and test methods (ISO 23162:2021)
EN ISO 17511:2021	2 June 2021	*In vitro* diagnostic medical devices - Requirements for establishing metrological traceability of values assigned to calibrators, trueness control materials and human samples (ISO 17511:2020)
EN ISO 23118:2021	2 June 2021	Molecular *in vitro* diagnostic examinations - Specifications for pre-examination processes in metabolomics in urine, venous blood serum and plasma (ISO 23118:2021)
EN ISO 20184-3:2021	26 May 2021	Molecular *in vitro* diagnostic examinations - Specifications for pre-examination processes for frozen tissue - Part 3: Isolated DNA (ISO 20184-3:2021)
CEN/TS 17626:2021	5 May 2021	Molecular *in vitro* diagnostic examinations - Specifications for pre-examination processes for human specimen - Isolated microbiome DNA
EN ISO 20776-1:2020	1 July 2020	Susceptibility testing of infectious agents and evaluation of performance of antimicrobial susceptibility test devices - Part 1: Broth micro-dilution References method for testing the *in vitro* activity of antimicrobial agents against rapidly growing aerobic bacteria involved in infectious diseases (ISO 20776-1:2019, including Corrected version 2019-12)
EN ISO 22367:2020	11 March 2020	Medical laboratories - Application of risk management to medical laboratories (ISO 22367:2020)
CEN/TS 17390-1:2020	22 January 2020	Molecular *in vitro* diagnostic examinations - Specifications for pre-examination processes for circulating tumor cells (CTCs) in venous whole blood - Part 1: Isolated RNA
CEN/TS 17390-2:2020	22 January 2020	Molecular *in vitro* diagnostic examinations - Specifications for pre-examination processes for circulating tumor cells (CTCs) in venous whole blood - Part 2: Isolated DNA
CEN/TS 17390-3:2020	22 January 2020	Molecular *in vitro* diagnostic examinations - Specifications for pre-examination processes for circulating tumor cells (CTCs) in venous whole blood - Part 3: Preparations for analytical CTC staining
EN ISO 20186-3:2019	23 October 2019	Molecular *in-vitro* diagnostic examinations - Specifications for pre-examination processes for venous whole blood - Part 3: Isolated circulating cell free DNA from plasma (ISO 20186-3:2019)
EN ISO 20186-1:2019	27 March 2019	Molecular *in vitro* diagnostic examinations - Specifications for pre-examination processes for venous whole blood - Part 1: Isolated cellular RNA (ISO 20186-1:2019)
EN ISO 20186-2:2019	27 March 2019	Molecular *in vitro* diagnostic examinations - Specifications for pre-examination processes for venous whole blood - Part 2: Isolated genomic DNA (ISO 20186-2:2019)
EN ISO 15195:2019	6 February 2019	Laboratory medicine - Requirements for the competence of calibration laboratories using References measurement procedures (ISO 15195:2018)
EN ISO 20166-3:2019	23 January 2019	Molecular *in vitro* diagnostic examinations - Specifications for pre-examination processes for formalin-fixed and paraffin-embedded (FFPE) tissue - Part 3: Isolated DNA (ISO 20166-3:2018)
EN ISO 20166-2:2018	19 December 2018	Molecular *in vitro* diagnostic examinations - Specifications for pre-examinations processes for formalin-fixed and paraffin-embedded (FFPE) tissue - Part 2: Isolated proteins (ISO 20166-2:2018)
EN ISO 20166-1:2018	19 December 2018	Molecular *in vitro* diagnostic examinations - Specifications for pre-examination processes for formalin-fixed and paraffin-embedded (FFPE) tissue - Part 1: Isolated RNA (ISO 20166-1:2018)
EN ISO 20184-1:2018	19 December 2018	Molecular *in vitro* diagnostic examinations - Specifications for pre-examination processes for frozen tissue - Part 1: Isolated RNA (ISO 20184-1:2018)
EN ISO 20184-2:2018	12 December 2018	Molecular *in vitro* diagnostic examinations - Specifications for pre-examination processes for frozen tissue - Part 2: Isolated proteins (ISO 20184-2:2018)
EN ISO 6710:2017	6 September 2017	Single-use containers for human venous blood specimen collection (ISO 6710:2017)
EN ISO 22870:2016	30 November 2016	Point-of-care testing (POCT) - Requirements for quality and competence (ISO 22870:2016)
EN ISO 15197:2015	10 June 2015	*In vitro* diagnostic test systems - Requirements for blood-glucose monitoring systems for self-testing in managing diabetes mellitus (ISO 15197:2013)
EN ISO 23640:2015	10 June 2015	*In vitro* diagnostic medical devices - Evaluation of stability of *in vitro* diagnostic reagents (ISO 23640:2011)
EN ISO 19001:2013	20 March 2013	*In vitro* diagnostic medical devices - Information supplied by the manufacturer with *in vitro* diagnostic reagents for staining in biology (ISO 19001:2013)
EN ISO 15189:2012	1 November 2012	Medical laboratories - Requirements for quality and competence (ISO 15189:2012, Corrected version 2014-08-15)
EN ISO 18113-5:2011	19 October 2011	*In vitro* diagnostic medical devices - Information supplied by the manufacturer (labelling) - Part 5: *In vitro* diagnostic instruments for self-testing (ISO 18113-5:2009)
EN ISO 18113-2:2011	19 October 2011	*In vitro* diagnostic medical devices - Information supplied by the manufacturer (labelling) - Part 2: *In vitro* diagnostic reagents for professional use (ISO 18113-2:2009)
EN ISO 18113-3:2011	19 October 2011	*In vitro* diagnostic medical devices - Information supplied by the manufacturer (labelling) - Part 3: *In vitro* diagnostic instruments for professional use (ISO 18113-3:2009)
EN ISO 18113-4:2011	19 October 2011	*In vitro* diagnostic medical devices - Information supplied by the manufacturer (labelling) - Part 4: *In vitro* diagnostic reagents for self-testing (ISO 18113-4:2009)
EN ISO 18113-1:2011	19 October 2011	*In vitro* diagnostic medical devices - Information supplied by the manufacturer (labelling) - Part 1: Terms, definitions and general requirements (ISO 18113-1:2009)
EN ISO 15193:2009	1 May 2009	*In vitro* diagnostic medical devices - Measurement of quantities in samples of biological origin - Requirements for content and presentation of References measurement procedures (ISO 15193:2009)
EN ISO 15194:2009	1 May 2009	*In vitro* diagnostic medical devices - Measurement of quantities in samples of biological origin - Requirements for certified References materials and the content of supporting documentation (ISO 15194:2009)
EN 14136:2004	19 May 2004	Use of external quality assessment schemes in the assessment of the performance of *in vitro* diagnostic examination procedures
EN ISO 18153:2003	15 August 2003	*In vitro* diagnostic medical devices - Measurement of quantities in biological samples - Metrological traceability of values for catalytic concentration of enzymes assigned to calibrators and control materials (ISO 18153:2003)
EN 13975:2003	19 March 2003	Sampling procedures used for acceptance testing of *in vitro* diagnostic medical devices - Statistical aspects
EN 13612:2002/AC:2002	18 December 2002	Performance evaluation of *in vitro* diagnostic medical devices
EN 13641:2002	8 May 2002	Elimination or reduction of risk of infection related to *in vitro* diagnostic reagents
EN 13532:2002	17 April 2002	General requirements for *in vitro* diagnostic medical devices for self-testing
EN 13612:2002	20 March 2002	Performance evaluation of *in vitro* diagnostic medical devices
EN 12322:1999/A1:2001	24 October 2001	*In vitro* diagnostic medical devices - Culture media for microbiology - Performance criteria for culture media
EN 12322:1999	21 April 1999	*In vitro* diagnostic medical devices - Culture media for microbiology - Performance criteria for culture media
EN 1659:1996	20 November 1996	*In vitro* diagnostic systems - Culture media for microbiology - Terms and definitions

## Data Availability

Requests to access the datasets should be directed to B4M= ED@ieo.it.
